# Vascular endothelial growth factor encoded by Parapoxviruses can regulate metabolism and survival of triple negative breast cancer cells

**DOI:** 10.1038/s41419-020-03203-4

**Published:** 2020-11-20

**Authors:** Dipayan Bose, Sagarika Banerjee, Rajnish Kumar Singh, Lyn M. Wise, Erle S. Robertson

**Affiliations:** 1 Department of Otorhinolaryngology-Head and Neck Surgery, Perelman School of Medicine, Tumor Virology Program, Abramson Cancer Center, University of Pennsylvania, Philadelphia, PA 19104 USA; 2 Department of Pharmacology and Toxicology, School of Biomedical Sciences, University of Otago, Dunedin, New Zealand

**Keywords:** Cancer, Cell biology

## Abstract

Dysbiotic microbiomes are linked to many pathological outcomes including different metabolic disorders like diabetes, atherosclerosis and even cancer. Breast cancer is the second leading cause of cancer associated death in women, and triple negative breast cancer (TNBC) is the most aggressive type with major challenges for intervention. Previous reports suggested that Parapoxvirus signatures are one of the predominant dysbiotic viral signatures in TNBC. These viruses encode several genes that are homologs of human genes. In this study, we show that the VEGF homolog encoded by Parapoxviruses, can induce cell proliferation, and alter metabolism of breast cancer and normal breast cells, through alteration of MAPK-ERK and PI3K-AKT signaling. In addition, the activity of the transcription factor FoxO1 was altered by viral-encoded VEGF through activation of the PI3K-AKT pathway, leading to reprogramming of cellular metabolic gene expression. Therefore, this study provides new insights into the function of viral-encoded VEGFs, which promoted the growth of the breast cancer cells and imparted proliferative phenotype with altered metabolism in normal breast cells.

## Introduction

Breast cancer is one of the most frequent cancers affecting 1 in 8 women in the United States^1^. There are four major types of human breast cancers, and among them triple negative breast cancer (TNBC) is the most aggressive form with the poorest prognosis^2^. Over the last few decades many studies to develop a better understanding of the molecular pathways have been explored so that potential targets can be identified for the development of new therapeutics. The development of breast cancer, similar to other cancer types is also linked to lifestyle, ethnicity, genetic background, and obesity. However, recent studies have identified microbiome present in the tumor microenvironment as another potential contributor which plays a major role in cancer development^3,4^. It is also well-established that greater than 18% of human cancers are tightly linked to the presence of infectious microorganisms, which were demonstrated to play crucial roles in their pathogenesis. Chronic infections are also identified as contributors which have been linked to ongoing inflammatory responses^5,6^. It should also be noted that different microbial antigens and secretory factors can also play critical roles in driving cancer pathogenesis^7^.

The human body is colonized by microorganisms estimated to be equivalent in numbers to that of human cells. These are predominantly either commensals or mutualistic microorganisms^8^ . An imbalance in this microbial population results from colonization of pathogenic microorganisms, which leads to the establishment of a dysbiotic microbiome and initiation of different pathogenic activities. This dysregulation in the microbiome is also now accepted as one of the major drivers in cancer pathogenesis^9,10^.

Previously, we reported that TNBC samples are characterized by the presence of an array of different microbial agents, which included Arcanobacterium, Brevundimonas, Sphingobacteria, Providencia, Prevotella, Brucella, Escherichia, Actinomyces bacteria; viruses which included Herpes, Parapox, Flavivirus, Polyoma and Retrovirus families; Fungi namely Piedra, Foncecaea, Phialophora, and Paecilomyces. This study was performed on a cohort of 100 TNBC samples 17 matched controls and 20 non-matched controls^11^. Analyses of these tumor samples identified Parapoxvirus signatures as one of the most abundantly found viral signatures present^11^. These viral agents have been previously reported to encode homologs of human VEGF^12,13^. Three sub-groups of Parapoxviruses (ORFV, PCPV, and BPSV) encodes viral homologs of human VEGFs (VEGF-E), which bears significant polypeptide sequence homology with the human cellular VEGF-A (27%, 29, and 46% respectively)^14^. Human VEGF-A is produced by different kinds of cells including endothelial, hematopoietic, and stromal cells in response to hypoxia and has binding potential with three different receptors namely VEGFR1, 2 and 3^15^ whereas the viral VEGF-E is only reported to bind with human VEGFR2 and neuropilin1^16^, to initiate downstream cellular processes.

Cancer cells are characterized by their metabolic reprogramming which is required to provide the necessary energy requirement for its growth and survival. Instead of undergoing oxidative phosphorylation, they rapidly metabolize glucose in an anaerobic manner to produce lactic acid by the phenomenon known as the Warburg effect^17^. Cancer cells metabolize glucose at a faster rate by upregulating glycolysis and downregulating glucose assimilation and gluconeogenesis^18^. Similarly, the fatty acid and amino acid biosynthetic pathways are also dysregulated. Notably, the glutamine metabolism and fatty acid oxidation pathways are the most dramatically affected pathways in breast cancer^19,20^. There are numerous previous reports about small molecules which targets against arginase (ARG) and nitric oxide synthase (NOS)^21^, arginine-succinate lyase (ASL)^22^, and the branched-chain amino acid amino transferase (BCAT1)^23^ of the amino acid metabolism which are all expressed in higher concentrations in breast cancer. Fatty acid metabolic genes like fatty acid synthase (FAS)^24^, carnitine palmitoyl transferase I (CPT1)^25^, acetyl-CoA carboxylase-α (ACC)^26^, 3-hydroxy-3-methylglutaryl-CoA synthase (HMGCS)^27^, and acyl-CoA cholesterol acyl transferase (ACAT)^28^, are also highly expressed in different cancers. However, it is well documented that glycolysis and the Kreb’s cycle, not only controls the flow of carbon in carbohydrate metabolism, but can indirectly affect lipid and amino acid metabolism by modulating the availability of the internal carbon pool of the cell^19,29^.

TNBC is one of the deadliest types of breast cancer because of its aggressiveness and the lack of responsiveness to different lines of available treatments. TNBC are negative to human epidermal growth factor receptor 2 (HER), progesterone receptor (PR), and estrogen receptors (ER), but are typically positive for receptor tyrosine kinase (RTK) receptors^30,31^. Previous studies have suggested that epidermal growth factor (EGF) binds to its cognate receptor, epidermal growth factor receptor (EGFR) to initiate downstream signaling that leads to the synthesis of cellular VEGF, which, in turn, binds to VEGFR and promotes cell proliferation^32^. Besides angiogenesis and cell proliferation, VEGF is also linked to maintenance of cell survival through regulation of metabolism and autophagy^33^. Furthermore, the PI3K-AKT and ERK-MAPK are the two central pathways that are involved in modulating transcription of metabolic genes^34,35^, and, therefore, contribute to the regulation of cellular metabolism.

In this study, we have identified Parapoxvirus DNA elements as a potential signature for a dysbiotic microbiome, and the viral VEGF-E is one of the factors modulating altered metabolism and cellular proliferation in TNBC through activation of the PI3K-AKT and ERK-MAPK pathway. Moreover, we have demonstrated that VEGF-E can induce higher proliferation and increased metabolism in normal breast cells. Our findings strongly suggest that the VEGF-E encoded by these Parapoxviruses can also reprogram normal breast cells to a hyperproliferative state with altered metabolism similar to that of cancer cells. Our study provides further evidence of dysbiotic microbiomes and their encoded secretory antigens capable of reprogramming the tumor and the surrounding cells, and thus contributing to progression of disease.

## Materials and methods

### Human study samples

This study was performed on a cohort of 11 TNBC samples (EXTN) and their corresponding surrounding non-cancerous tissues or matched control (EXMC), and 10 tissue samples from healthy individuals as non-matched control (NC). The NC samples are breast tissues from normal individuals. No patient identifiers were available to the laboratory staff, and their use were approved by the institute (IRB protocol number 832857). All experiments with formalin-fixed paraffin-embedded tissues samples (FFPE) were performed according to relevant guidelines and regulations, which were approved by the institutional committee of the Perelman School of Medicine, University of Pennsylvania. No information was obtained related to the treatment regimens of these breast cancer patients. All these tissues were obtained as de-identified archived samples. Tumor samples were received either as 10 µm rolls or in 10 µm sections on slides. Pathology staff reviewed the case histories, confirmed the tumor types and demarcated the cancer cells. Proper care was taken during dissection of the samples to control for environmental contamination and to minimize the potential cross contamination between samples by sterilizing the blades and the field^36,37^.

### PathoChip design

The details of the PathoChip array have been described previously^11^. It comprises 60,000 probes that represents over 6000 different microorganisms (viruses, bacteria, fungi, and parasites) listed in GenBank. The arrays were manufactured by Agilent Technologies Inc. and contains eight replicate arrays per slide. Probes are of 60 nucleotide DNA oligomers that targets genomic regions of viruses, prokaryotic and eukaryotic microorganisms. The PathoChip technology, combined with PCR and capture-next-generation sequencing (NGS), is a valuable strategy for detecting and identifying pathogens in human cancers and infection-related pathologies.

The DNA and RNA were extracted from the samples and were subjected to whole-genome and transcriptome amplification (referred here as WTA) using the TransPlex Complete Whole Transcriptome Amplification Kit (Sigma-Aldrich, St. Louis, MO), using 50 ng each of RNA and DNA as input. The WTA were processed as described earlier^11^. The data were extracted from the Agilent SureScan G4900DA array scanner and were analyzed as described previously^11,36,37^. The R program was used to normalize the data and on the normalized signals *t*-test was applied to select probes significantly present in cancer samples by comparing cancer samples versus controls and to select probes significantly present in the breast cancer samples versus the controls.

PathoChip results were validated using specific primer pairs for the target microorganism. Quantitative PCR with cut-off of 35 cycles for each of amplicons were used for each of the primer pairs. Samples in which there is at least minimum amplification (above threshold values) are regarded as positive and all others were considered as negative.

The WTA prepared form the tissues samples were used as template for the real-time PCR when determining the expression of the selected metabolic genes. Primer sequences are provided in SI Tables 1–3.

### Cell lines, plasmids, antibodies, and inhibitors

The TNBC cell line (MDA-MB-231), and human mammary epithelial cells (HMEC) were purchased from ATCC and were maintained in recommended culture condition as per the ATCC instructions^38^. The viral VEGF-E homologs (PCPV-VEGF, ORFV-VEGF, and BPSV-VEGF) were a kind gift from Dr. L.M. Wise, University of Otago, Dunedin New Zealand^39^. Antibodies against GFP, PCNA, GAPDH, p-Erk, p-MEK, p-Raf, AKT, FoxO1, H2A were purchased from Santa Cruz Biotechnology Inc Dallas, TX, and ERK, PI3k110α, and p-AKT was purchased from Cell Signaling Technologies Inc. Danvers, MA and anti-BrdU antibody from Invitrogen, Thermo Fisher (SI Table 4). The secondary antibodies for western blot are IR conjugated and were from LI-COR Inc. Lincoln, NE, while for microscopy the secondary antibody is Alexa 594 conjugated purchased from Invitrogen Inc. Carlsbad, CA.

LY294002, an inhibitor for PI3K was purchased from Sigma Aldrich Inc. St. Louis, MO. MDA-MB-231 cells were treated with 50 µM and HMEC cells with 30 µM of LY294002 and incubated for 24 h before being used for assays.

### Lentiviral transduction and preparation of stable cell line

The pLVX-AcGFP Lentiviral plasmid was double digested with AccI and BamH1 and was used to clone the three viral VEGF-E homologs with a signal sequence fused with GFP at the C-terminal. For MDA-MB-231 and HMEC cells with VEGF-E stable expression, the VEGF-expressing plasmid pLVX-AcGFP-VEGF or vector alone, was individually co-transfected with Lentivirus package-expressing plasmids (Rev, vesicular stomatitis virus G [VSVG], and glycoprotein) into HEK293T cells to generate virus. The packaged viruses were used to individually transduce MDA-MB-231 and HMEC cells, with selection by 1.5 μg/ml puromycin for MDA-MB-231 and 1 μg/ml for HMEC cells until all the cells are positive to GFP . The expression of the GFP fused VEGF-E proteins were tested by western blot using antibody against GFP and immunofluorescence analysis for GFP signals.

### Western blot and confocal microscopy

Cell lysates were prepared from 10^6^ cells, and total proteins were isolated. 40 µg of proteins were loaded in each well and were resolved on SDS-polyacrylamide gels followed by wet transfer to nitrocellulose membrane. 5% skimmed milk/ BSA was used for blocking at room temperature for 1 h with gentle shaking. The membranes were incubated on primary antibodies for overnight at 4 °C followed by 30 min washing with TBST and probing with infrared (IR) dye conjugated secondary antibody. Membranes were scanned using an Odyssey scanner (LI-COR Inc., Lincoln, NE) for detection of specific antigens and the band intensities were measured using the ImageQuant, Odyssey software. The band intensities were normalized with that of the loading control (GAPDH) and the fold intensity values were represented below each blot.

For confocal microscopy, cells were plated over coverslips. Coverslips were washed with ice cold PBS and fixed with 4% paraformaldehyde for 30 mins at room temperature and perforated by 0.05% of TritonX-100 for 15 mins. Perforated cells were washed with PBS and incubated with primary antibody (1:150) overnight at 4 °C, followed by 30 min washing with PBST and probing with Alexa fluorophore-conjugated secondary antibody (1:300) for 1 h before being washed with PBST, stained with DAPI and fixed on glass slides. The images were observed using an Olympus F300 Confocal Microscope. Microscopic images were analyzed using ImageJ (Adobe Inc., San Jose, CA), and the co-localization co-efficient (Pearson’s co-efficient, r) was measured by the Coloc2 program of ImageJ, and the values were tabulated in graphical format. Each bar represents the value of at least 10 microscopic fields each from three independent experiments.

### Colony formation and cell proliferation assay

10^5^ transfected or non-transfected cells were plated with DMEM supplemented with 5% BGS in 10 cm dish and after 5 days cells were fixed with 4% paraformaldehyde, and stained with 0.05% of crystal violet solution overnight, and visualized using a BioRad Chemidoc Imaging system (BioRad Inc. Hercules, CA). The colony number and size were quantitated using ImageJ.

For cell proliferation assays, 10^6^ cells were washed with PBS and treated with 10 µM BrdU in complete growth medium and incubated for 24 h at 37 °C in a CO_2_ incubator. Cells were washed twice with PBS and fixed with 4% PFA and permeabilized with 0.05% of TritonX-100 for 15 min and washed with PBS before being stained with anti-BrdU antibodies conjugated with PE fluorophore^40^ and the fluorescence was checked with a flow cytometer (Becton-Dickinson Inc., San Jose, CA) and the data were analyzed by FlowJo Software (Treestar, Inc., San Carlos, CA). The Mean fluorescence intensity (MFI) was calculated and results are represented in the provided graphs.

For MTT assay 10^4^ cells were plated in each well of 96 well plate and grown for overnight. The exhausted media is discarded and the cells are treated with 100 µl of 5 mg/ml of MTT solution prepared in serum free media for 4 h at 37 °C in dark and 100 µl of DMSO is added to solubilize and dissolve the formazan formed and the absorbance was checked at 590 nm in Cytation 5 (BioTek, VT, United States) and plotted in Graphpad Prism.

### Isolation of nuclear proteins

Nuclear lysate was prepared according to the method previously described^41^. 10^7^ cells are washed with PBS, dislodged and pelleted by centrifugation and resuspended in the cell lysis buffer [10 mM HEPES; pH 7.5, 10 mM KCl, 0.1 mM EDTA, 1 mM dithiothreitol (DTT), 0.5% Nonidet-40 and 0.5 mM PMSF along with the protease inhibitor cocktail (Sigma)] and allowed to swell on ice for 15–20 min with intermittent mixing. Tubes are vortexed to disrupt cell membranes and then centrifuged at 12,000 × *g* at 4 °C for 10 min.

The pelleted nuclei were washed 3 times with the cell lysis buffer and resuspended in the nuclear extraction buffer containing 20 mM HEPES (pH 7.5), 400 mM NaCl, 1 mM EDTA, 1 mM DTT, 1 mM PMSF with protease inhibitor cocktail and incubated in ice for 30 min. The nuclear extract was collected by centrifugation at 12,000 × *g* for 15 min at 4 °C.

### RNA isolation, cDNA preparation, and real-time PCR

RNA isolation was performed as per the standard method using TRizol reagent (Ambion, Grand Island, NY) and phenol-chloroform extraction. 2 μg of total RNA was used to prepare the cDNA by a random priming method using the Superscript cDNA synthesis kit (Applied Biosystems Inc., Foster City, CA). The cDNA were diluted 10 times with 1 μl of the diluted cDNA used for each 10 μl volume of PCR reaction, with Power SYBR green PCR reagent (Applied Biosystems Inc., Carlsbad, CA) and Step One Plus, or Quant Studio PCR system (Applied Biosystems Inc., Carlsbad, CA). All the primers were designed from NCBI and were purchased from Integrated DNA Technologies Inc. (Coralville, IA). All real-time PCR assays were performed in duplicates, with at least two experimental repeats for each gene. The heat maps were generated by using the online Heatmapper software^42^. The sequence of primers used for the PCR reactions are provided in SI Tables 1–3.

## Results

### Analysis of microbial population associated with triple negative breast cancer

**Fig. 1:**
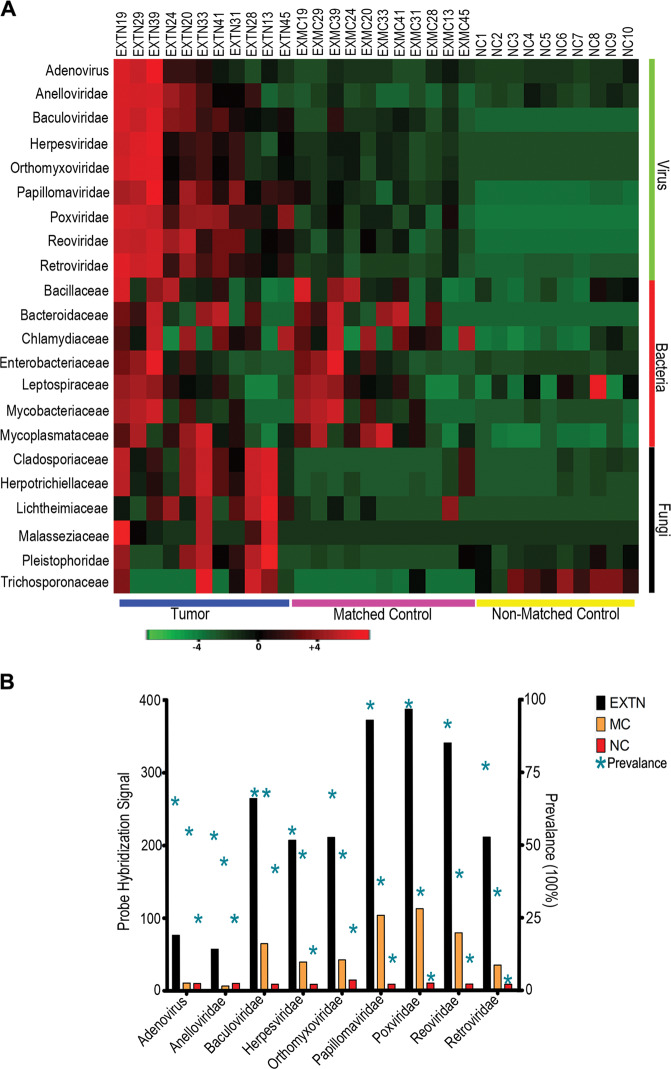
Detection of viral, bacterial, and fungal signatures in the tumor samples (EXTN), its corresponding matched control (EXMC) and non-matched control (NC). **A** Signals for microbial signatures were detected in the EXTN, EXMC, and NC and are shown as heat maps of microorganism (*y*-axis) hybridized to the tumor samples and both matched (MC) and non-matched control (NC) samples (*x*-axis). The matched controls were obtained from the adjacent normal breast tissues of the breast cancer samples. **B** Total hybridization signal for the most abundant viruses for all the tumor samples, matched control and the non- matched control were plotted. The prevalence of each of the virus in the different samples were represented with asterisk above the bar.

Using Pathochip technology^11,43^ we screened 11 TNBC samples (EXTN) and their corresponding adjacent normal tissues as matched control (EXMC), and 10 healthy breast tissues samples as non-matched control (NC) for the presence of nucleic acids from a wide range of different microorganisms. Unique and common microbial signatures associated with the TNBC samples were compared to that of the matched and non-matched control and was tabulated in the form of a heat map (Fig. 1A). The probes which had a significantly higher hybridization signal (*p*-value < 0.05, log2 fold change in hybridization signal > 1) were regarded as positive.

In this study, we analyzed the Pathochip data which identified 9 viruses, 7 bacteria, and 6 fungi with the highest hybridization signal and are most prevalent in the TNBC samples compared to the non-matched control. Among the 9 virus signatures identified to be unique in the tumor samples, Pox, Papilloma, and the Reo virus families were the most abundant with more than 90% prevalence among the tumor tissues, compared to 35-40% in matched control and less than 10% in non-matched control. Our Pathochip analyses showed that signatures for Poxviruses had the highest hybridization signals followed by Papilloma viruses (Fig. 1B).

To validate the presence or absence of Poxvirus in the tissue samples, primers specific for the three different strains of Poxvirus (SI Table 1) were used and real-time PCR was performed. DNA isolated from the strains of virus were used as positive control and DNA from MDA-MB-231 cells were used as negative control. The results showed that only two tumor sample (EXTN28, 33) was negative for both ORFV and BPSV signatures while EXTN20, 41, 45 was negative for PCPV signatures (SI Fig. 1A). In matched controls 4, 5, and 7 samples among a total of 11 samples were found to be negative for signatures of ORFV, PCPV, and BPSV respectively (SI Fig. 1B). Among the non-matched control all samples were negative for Poxvirus genome signatures except NC3, which was found to be positive for BPSV genomic DNA (SI Fig. 1C).

### Detection of viral-encoded VEGF-E homolog in tissue samples

The presence of VEGF-E, which represents homologs of human VEGFA encoded by the parapox virus^39,44^ in the tissue samples was confirmed by real-time PCR using primers specific for the three types of Poxviruses-encoded VEGF-E homologs, and human VEGFA was used as positive control. The real-time PCR analyses are represented as a heat map in (SI Fig. 2). Among the tumor samples, two samples (EXTN33 and EXTN28) were ORFV-VEGF-E negative, three samples (EXTN28, 39, 33) were BPSV-VEGF-E negative and four samples (EXTN20, 24, 45 and 41) were PCPV-VEGF negative. All other amplifications were positive for either ORFV, BPSV and PCPV (SI Fig. 2, left panel). Analysis of the matched controls showed that four samples (EXMC 28, 29, 31, and 33) were ORFV-VEGF-E negative, six samples (EXMC 13,20, 39, 45, 33, 31) were PCPV-VEGF-E negative, and six samples (EXMC 19, 28, 20, 29, 39, 31, and 33) were BPSV-VEGF negative. All others have positive signals for the poxvirus VEGF-E homologs (SI Fig. 2, middle panel). For the Non-matched control samples, only one sample (NC3) was found to be positive for BPSV-VEGF, and none of the others were positive for ORFV-VEGF-E or PCPV-VEGF-E (SI Fig. 2, right panel). All samples, including the EXTN, EXMC, and NC were positive for the presence of human VEGFA transcripts as would be expected (SI Fig. 2). Therefore, our results indicate that more than 80% of the tumor samples are positive for VEGF-E as compared to approximately 60% for the matched controls. Importantly, only 10% of the non-matched tissues were positive for the VEGF-E.

### Parapoxvirus-encoded VEGF-E can modulate metabolic pathways

One hallmark of all tumors is altered metabolism with upregulated glucose uptake and anaerobic respiration (glycolysis), and subsequent entry into the TCA cycle which is required for production of the available ATP and NADPH^45^. The amino acid glutamine is an essential amino acid that links carbohydrate metabolism to nucleotide biosynthesis. Hyper-proliferating cancer cells requires a steady supply of nucleotides and require 10–100 times more glutamine compared to normal cells^46^. Similarly, higher levels of de novo fatty acid synthesis from acetyl CoA, and mitochondrial beta-oxidation are also observed in the cancer cells compared to normal cells^29,47^.

**Fig. 2:**
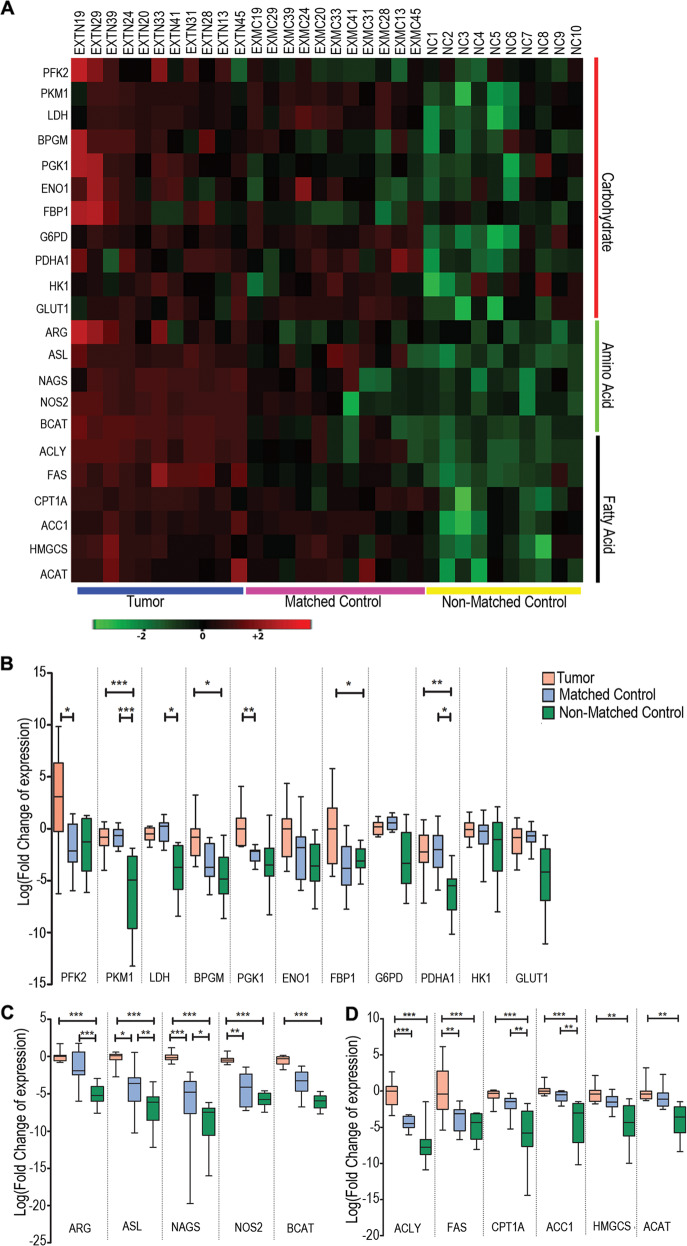
Differential expression of the metabolic genes in tumor samples compared to the matched and non-matched control. The heat maps represent the logarithmic values of the expression levels of the different metabolic genes. **A** The fold change of the expression of each of the genes were represented in the heat maps. A set of 11 carbohydrate, 5 amino acid, and 6 lipid metabolism genes were examined. **B**–**D** The logarithmic values of the fold change of expression of all the genes of carbohydrate, amino acid, and fatty acid metabolism for each of the groups i.e., tumor, matched control, and non-matched control were represented as bars and are compared among themselves. The real-time PCR experiments were performed in duplicates, with two experimental repeats for each gene. *P < 0.05; **P < 0.005; ***P < 0.001.

We selected 11 genes linked to carbohydrate metabolism, 5 genes linked to amino acid metabolism, and 6 genes involved in fatty acid metabolism and examined their transcriptional expression in the tissue samples. These genes were previously reported to be altered when examined in several cancers^48^. Our results showed that the tumor samples were found to have higher expression of these genes compared to that of the matched controls whereas the non-matched controls showed minimal expression (Fig. 2A–D).

**Fig. 3:**
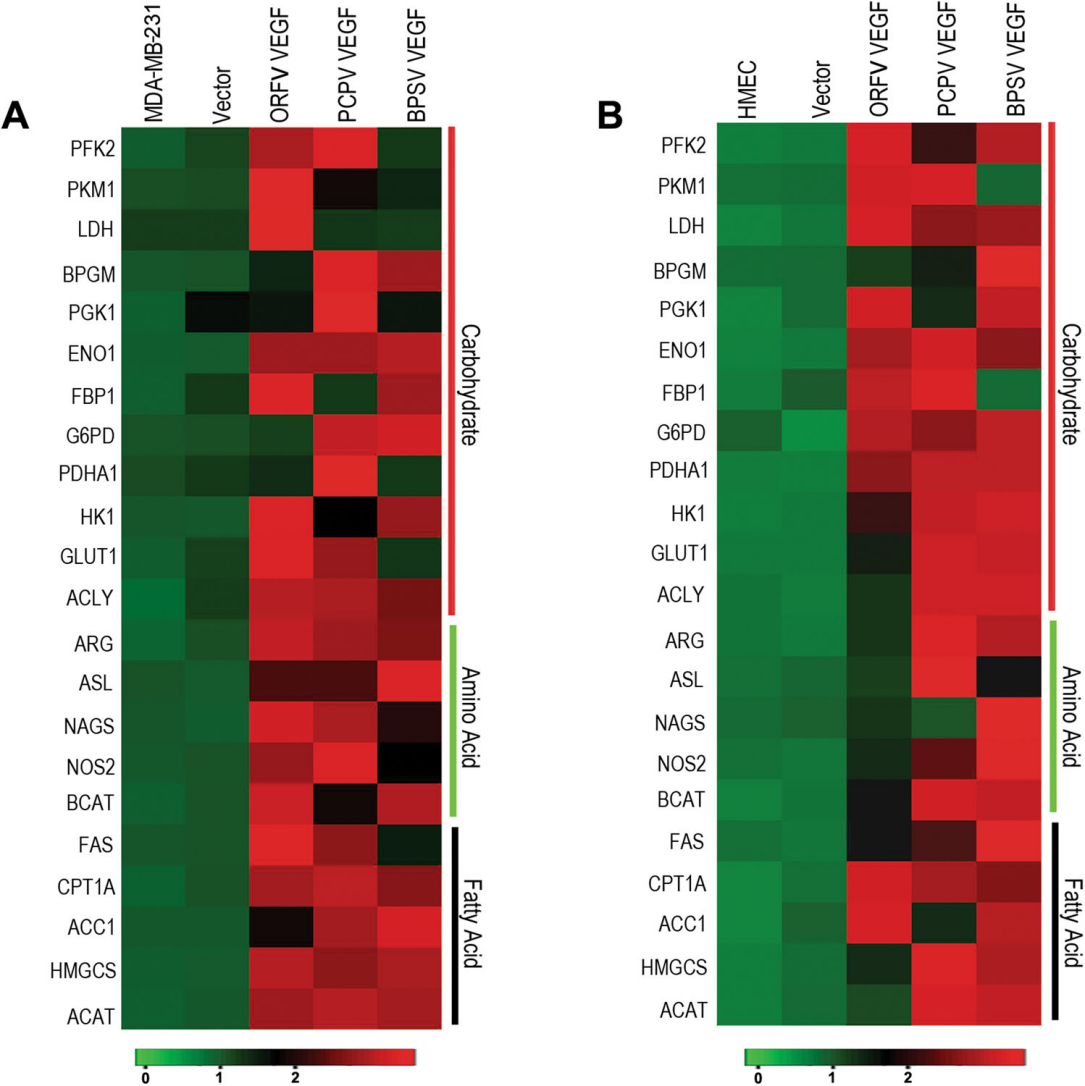
Differential expression of the metabolic genes in VEGF transfected MDA-MB-231 and HMEC cells compared to the mock transfected cells. **A** MDA-MB-231 or **B** HMEC cells were transfected with empty vector, ORFV VEGF-E, PCPV VEGF-E or BPSV VEGF-E and relative expression of the metabolic genes were calculated using real-time PCR as compared to the non-transfected cells. The heat maps represent the logarithmic values of the fold change of the different metabolic genes compared to the non-transfected cells and normalized with 18S rRNA. A set of 11 carbohydrate, 5 amino acid, and 6 lipid metabolism genes were examined. The real-time PCR experiments were performed in duplicates, with two experimental repeats for each gene.

Tumor samples have been shown to have a variety of different microbiome signatures compared to their matched control and non-matched normal tissue controls^11,36,37^. To confirm the involvement of the Poxvirus-encoded VEGF-E in altered metabolism, three VEGF-E (ORFV-VEGF, PCPV-VEGF, and BPSV-VEGF) were cloned in Lentiviral plasmids with GFP (pLVX AcGFP) (SI Fig. 3A). All constructs were confirmed by sequencing (data not shown). The expressions were further determined at the protein level by western blot to monitor the level of the GFP-fused protein using GFP specific antibodies (SI Fig. 3B). The viral VEGFs were transduced by lentiviral vectors into triple negative breast cancer cells (MDA-MB-231) and the normal breast cells (HMEC) in vitro. The results showed upregulation of the selected metabolic genes in both cell types (Fig. 3A, B). Importantly, the fold increase in the levels of expression of the metabolic genes in stably transfected HMEC was much higher than that of the transfected MDA-MB-231 cells as compared to the non-transfected cells (SI Table 5). MDA-MB-231 is a TNBC cell line, which already has upregulated expression of these metabolic genes while the HMEC cells are normal breast epithelial cells^49^. Therefore, transfection of normal breast cells with viral-encoded VEGF homologs have a more pronounced effect on normal breast HMEC cells compared to that of the MDA-MB-231 cells (Fig. 3 and SI Table 5).

### Viral-encoded VEGF homologs can induce proliferation of breast epithelial cells

**Fig. 4:**
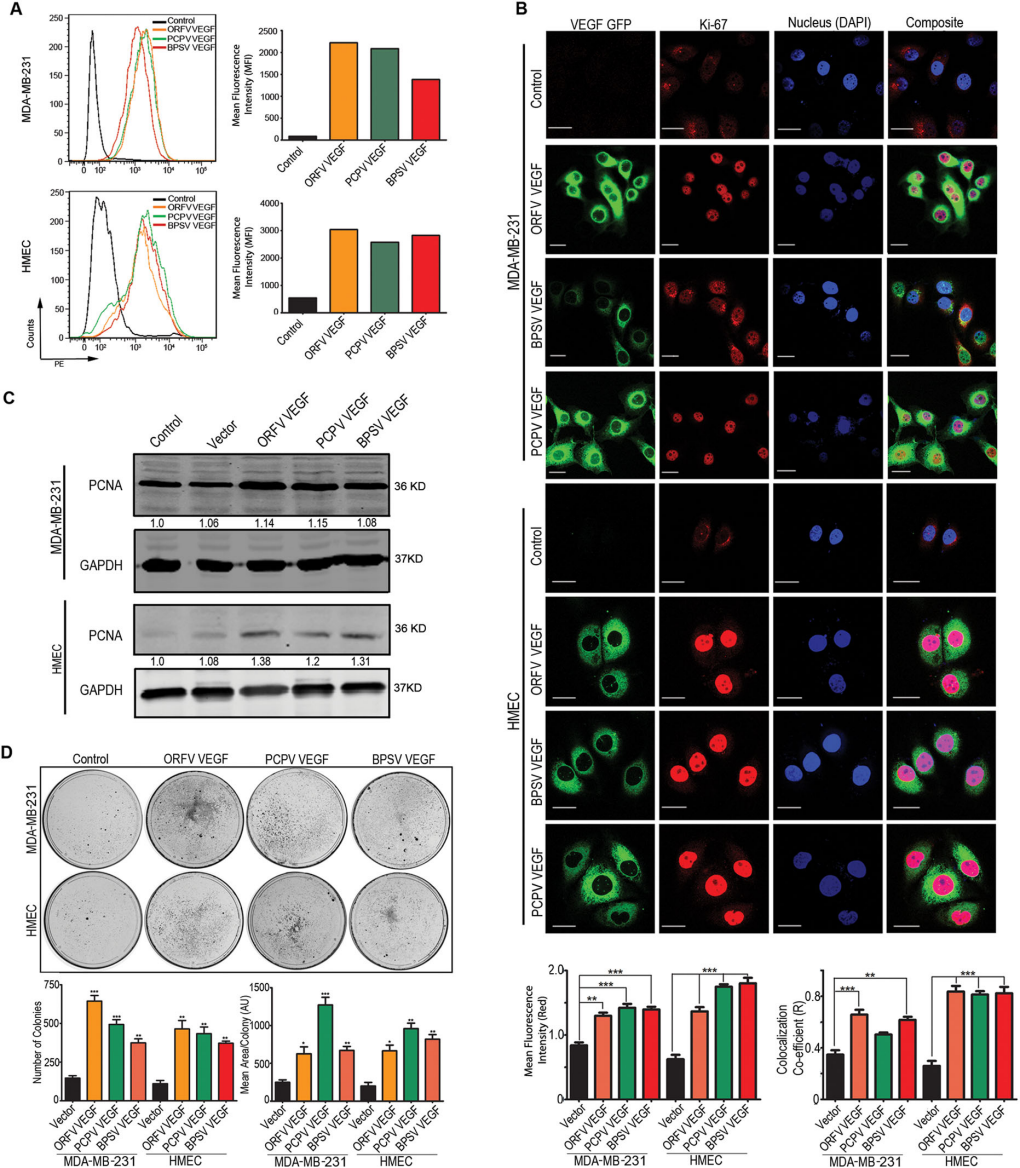
Proliferation of cell transduced with VEGF-E homologs encoded by Parapox viruses. Stable transfected MDA-MB-231 and HMEC cells with ORFV, PCPV, and BPSV VEGF-E were used for proliferation assay. **A** Cells were treated with BrdU and after 24 h stained with PE conjugated anti-BrdU antibody and the change of mean fluorescence intensity (MFI) were compared to that of the untransfected cells. The graphical representation showing the change of MFI for 24 h. **B** Microscopic study was performed to analyze the expression and localization of a proliferative marker Ki67. VEGF-E expression was monitored by the expression of the fused GFP protein. Expression of Ki67 and its localization in the nucleus was studied microscopically and results were analyzed using ImageJ and the change of expression of Ki67 is represented as change of mean fluorescence intensity and the localization in the nucleus as Pearson’s co-localization co-efficient (R). The results represent the values of 5 different fields and two independent experiments. *P < 0.05; **P < 0.005; ***P < 0.001. Each Scale bar is 20 µm. **C** Expression level of PCNA was studies by western blot. The band intensity was measured and normalized by the expression of GAPDH using the Odyssey software 3.0 and normalized with GAPDH. **D** Colony formation assay was performed to study the cell proliferation and growth. 10^5^ cells were plated in 10 cm cell culture dish and were grown for 5 days before being fixed and stained with 0.005% crystal violet. The colony size and number were calculated by ImageJ.

Alteration of cellular proliferation was investigated using Bromouridine (BrdU) staining. MDA-MB-231 and HMEC cells were transduced with Lentivurus containing the viral-encoded VEGF homologs and was treated with BrdU for 24 h then fixed and perforated and stained with anti-BrdU antibody tagged with PE fluorophore and analyzed to determine their degree of proliferation (Fig. 4A).Viral-encoded VEGF homologs expressing cells showed much higher mean fluorescent intensity (MFI) after compared to the empty vector transfected control (Fig. 4A). This demonstrated a higher proliferation rate compared to the mock transfected cells. In the case of HMEC cells, the change in mean fluorescence intensity (MFI) was, therefore, substantially greater when compared to the MDA-MB-231 cells (Fig. 4A). This variation may be because MDA-MB-231 cells are already transformed and therefore have a higher proliferation rate compared to the HMEC cells. Thus, the alteration in proliferation of HMEC was more significant when compared to the MDA-MB-231 cells which are already hyperproliferative due to their transformed state.

To provide additional evidence in support of these results, we used two different markers of proliferation, Ki67 and PCNA. Confocal microscopy of Ki67 showed higher expression, and its localization in the nucleus of cells expressing viral-encoded VEGF-E homologs compared to the non-transfected cells. The signals were plotted for mean fluorescence intensity and co-localization with the nucleus. Graphs showing the results are shown on right panels (Fig. 4B, top right panel). The expression levels of PCNA was also found to increase significantly in the transfected cells as demonstrated by western blot analysis for both cell lines stably expressing the VEGF homologs (Fig. 4C).

Colony formation assays were also performed to assess the proliferation rate and the ability of cells to form colonies. 10^5^ cells were plated, and colony formation was evaluated after 7 days. We observed a significant increase in the number and size of colonies in the cells stably expressing the VEGF homologs compared to the non-transfected cells indicating higher proliferations and increased oncogenic activities of these cells (Fig. 4D).

To further confirm that the secreted VEGF-E is responsible for the altered cellular growth, the VEGF-E or mock stable transfected cells (MDA-MB-231 and HMEC) were cultured and after 24 h the cell supernatant was collected and it was used at a ratio of 1:1 with fresh media to culture MDA-MB-231 and HMEC (without VEGF-E) and the proliferation was measured by MTT assay (SI Fig. 4A) and the data clearly shows that there is 1.3–1.8 fold more cellular proliferation in presence of the viral VEGF-E containing media compared to mock for MDA-MB-231 and 1.2–1.7 fold compared to mock for the HMEC cells suggesting the secreted VEGF-E is responsible for the cellular proliferation.

To further confirm that the secreted VEGF-E is responsible for the proliferation, the VEGF-E or mock stable transfected cells (MDA-MB-231 and HMEC) were cultured and after 24 h and the cell supernatant was collected and treated with protein A/G tagged anti-GFP neutralizing antibody for 4 h and centrifuged to precipitate the GFP fused viral VEGF-E protein from the supernatant and is used to treat MDA-MB231 and HMEC cells. The proliferation was measured by MTT assay (SI Fig. 4B). No significant change in proliferation observed between the cells treated with mock transfected media and viral VEGF transfected media. This finding clearly points out that the proliferative role of the viral VEGF-E.

### Viral-encoded VEGF-E can mediate PI3K-AKT pathway activation

**Fig. 5:**
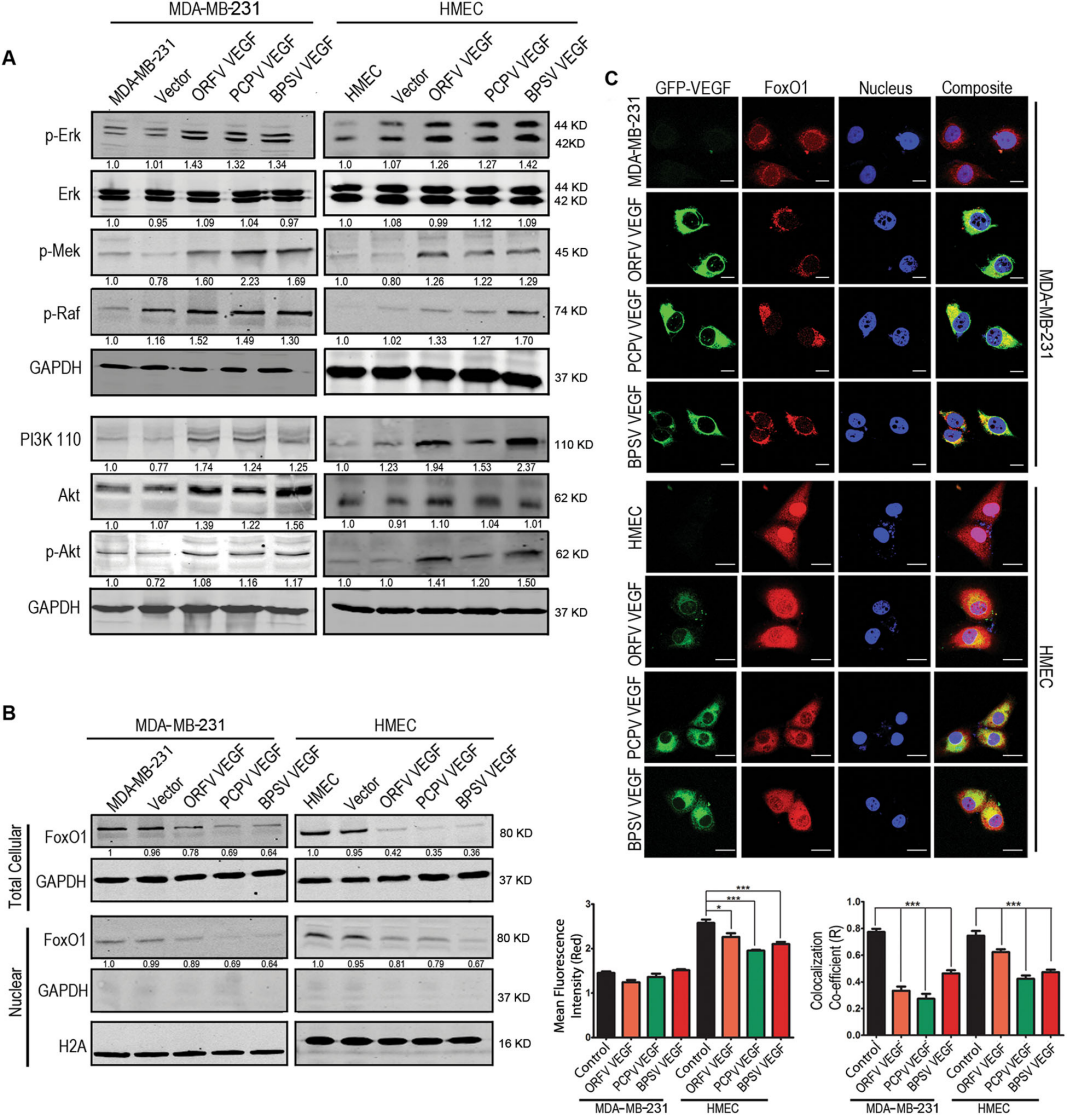
Activation of PI3K-AKT and MAPK-ERK pathway and alteration of FoxO1 expression. Stable transfects of MDA-MB-231 and HMEC cells with ORFV, PCPV, and BPSV VEGF-E showing higher levels of PI3K-AKT and MAPK-ERK pathway and altered expression and localization of FoxO1. **A** Western blot analysis of the PI3K-AKT and MAPK-ERK pathway proteins was performed and were normalized with that of GAPDH. The band intensities were measured by Odyssey software 3.0. **B** Western blot analysis of FoxO1 protein was performed with total cell lysate or with the nuclear lysate. H2A was used as nuclear loading control and for normalization while GAPDH is used to check cytoplasmic contamination. The band intensity were measured by Odyssey software 3.0. **C** Microscopic study to analyze the expression and localization of FoxO1 transcription factor. VEGF-E expression was monitored by the expression of the fused GFP protein. Expression of FoxO1 and its localization in the nucleus and cytoplasm was studied and the microscopic results were analyzed by ImageJ and the change of expression of FoxO1 is represented as change of mean fluorescence intensity and the localization in the nucleus as co-localization co-efficient (R). The results represent the values of 5 different fields and two independent experiments. *P* < 0.05; ***P < 0.001; ****P < 0.0001. Each Scale bar is 20 µm.

Previous reports have suggested that cellular VEGF, activated both the PI3K-AKT pathway and the ERK-MAPK pathway^50^. The PI3K pathway includes AKT, which is known to enhance cell survival by stimulating cell proliferation and angiogenesis^51^. Similarly, human VEGF has also been shown to activate the MAPK-ERK pathway and promotes survival of cells^52^. Therefore, we examined the expression of a number of downstream proteins of these pathways. Our results showed that the p-ERK, p-MEK and p-RAF of the ERK-MAPK pathway, and p110α and p-AKT of the PI3K-AKT pathway were found to be upregulated in both MDA-MB-231 and HMEC cells stably expressing the VEGF-E homologs from the Lentivirus when compared to the non-transfected cells (Fig. 5A). This suggests that the VEGF-E homologs can mediate the upregulation of these signaling pathways.

### Involvement of FoxO1 in metabolic gene expression

The Fork head transcription factor (Fox or FHKR) is a group of four different transcription factors that are mainly involved in a number of pathophysiological processes like ageing, cancer, altered metabolism and cell death^53–55^. FoxO1 is a member of the Fox family, which is mainly related to expression of different metabolic genes in response to insulin in patients suffering from diabetics^56,57^. However, recent reports also indicates that phosphorylation or methylation of FoxO1 sequesters it from the nucleus to the cytoplasm and can contribute to proliferation and altered metabolism in cancer^58–60^. In our study, we were interested in examining the levels of FoxO1 and its localization in cells stably expressing the VEGF-E homologs. We found that both the total cellular and nuclear FoxO1 levels were reduced in cells expressing the VEGF-E homologs compared to the non-transfected or mock transfected cells (Fig. 5B). To further ascertain the localization of FoxO1, we performed fluorescence microscopic study which showed that expression of the VEGF-E homologs not only reduced the levels of FoxO1, but also led to translocation of FoxO1 from the nucleus to the cytoplasm as evident from the co-localization co-efficient (Fig. 5C).

### VEGF-E homologs alters FoxO1 through PI3K-AKT pathway

**Fig. 6:**
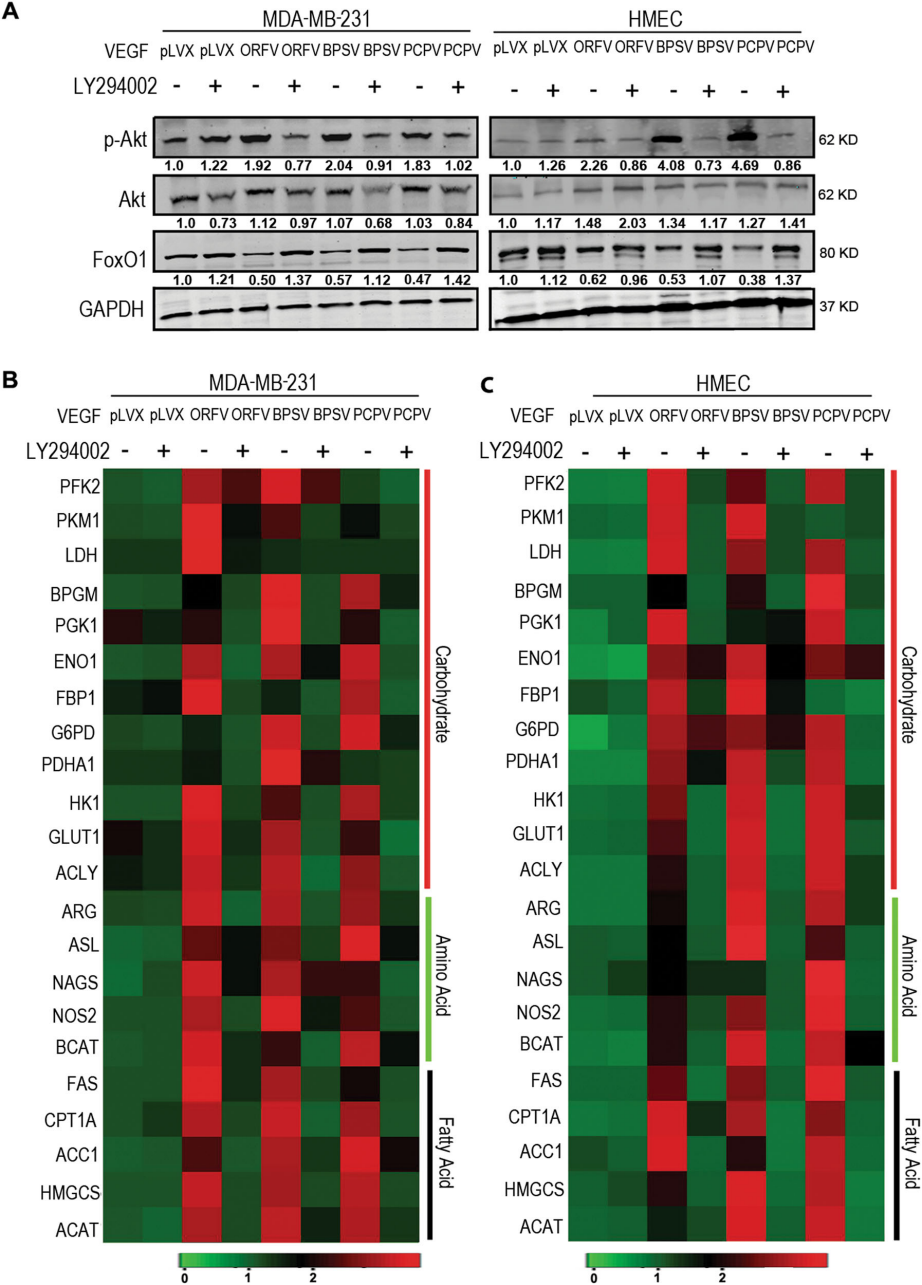
Effect of LY294002 on the FoxO1 expression and on the expression of metabolic genes. Transfected or non-transfected cells were treated with LY294002 (50 µM for MDA-MB-231 cells and 30 µM for HMEC cells) and its downstream targets were monitored. **A** Western blot analysis was performed to analyze the effect of LY294002 on the levels of phosphorylation of AKT and FoxO1 and were compared with that of inhibitor untreated cells. The band intensity was measured by Odyssey software 3.0 and normalization was done with GAPDH. **B**, **C** Quantitative PCR was performed to check the expression pattern of the metabolic genes in presence or absence of LY294002 in mock or VEGF-E transfected MDA-MB-231 and HMEC cells. The heat maps represent the logarithmic values of the fold change of the different metabolic genes compared to the non-transfected cells.

To further support the involvement of the PI3K pathway, we used LY294002, an inhibitor of the PI3K pathway^61,62^. LY294002 was found to inhibit the activity of PI3K as evident from the western blot analyses (Fig. 6A). PI3K activates AKT through phosphorylation at the serine 473 residue^63^. LY294002 significantly decreased the p-AKT levels suggesting inhibition of the pathway. We observed that the levels of FoxO1 increased in LY294002 treated cells compared to the cells expressing the VEGF-E homologs and suggest that inhibition of the PI3K pathway can led to accumulation of the total cellular FoxO1 (Fig. 6A).

As FoxO1 is related to expression of the metabolic genes, we investigated the mRNA levels of the different metabolic genes in LY294002 treated cells. LY294002 treatment led to the downregulation of mRNA expression of a range of metabolic genes in cells expressing the VEGF-E homologs (Fig. 6B). These results clearly suggest that inhibition of the PI3K pathway in the cells expressing the VEGF-E homologs reversed the phenotype to that of the non-transfected cells and thus links the PI3K pathway to metabolic gene expression in the presence of the viral-encoded VEGF-E homologs.

## Discussion

The microbiota in combination with the host becomes a super-organism in which this symbiotic relationship is mutually beneficial^64^. However, a change in the host microenvironment promotes implantation and proliferation of dysbiotic microorganisms that may contribute to a range of different pathologies. These dysbiotic microorganisms has been linked to, and is being regarded as causative agents of a number of different pathophysiological conditions, including diabetes, cancer, atherosclerosis, and cardiac disorders^37,65–67^. The dysbiotic microbiota can also directly interact with cellular signaling pathways, or they can be activated by metabolites secreted by the associated microorganisms to initiate and maintain the pathogenic phenotype^68^. Dysbiosis in the gut microbiome has been linked to chronic inflammation that can result in cancers of the gastrointestinal track^69^. Moreover, there are also reports about abnormal gut microbiome, which contributes to the onset of breast cancer^70,71^. However, there are no clear reports about the dysbiotic breast microbiome and its relationship to cancer progression.

Cancer cells are highly proliferating and, therefore, require a continuous supply of energy for their survival. To meet this high-energy demand, cancer cells have developed a different metabolic strategy compared to the normal cells^72^. TNBC are aggressive forms of breast cancer and due to the lack of the HER, ER, and PR receptors, most of the available hormonal therapies are thus ineffective.

There are few previous reports about dysbiotic microbiomes and their association with breast cancer^73^. Previous report from our lab was one of the pioneering study on TNBC that extensively identified different dysbiotic microbiomes^11^. In the present study, we have screened a new cohort of 11 TNBC samples and its corresponding matched controls and 10 non-matched controls using the previous protocol^43^. We found that dsybiotic Parapoxvirus signatures are the highest intensity of resident microbial genome signatures that are predominately found in the tumor samples compared to the matched control and almost absent in the non-matched control, which were validated by PCR using specific primers for the Parapoxvirus families. The abundance of Parapoxvirus genome signatures in TNBC samples is congruent with our previous study, which was based on a larger cohort of 100 samples^11^. We were also interested in investigating the status of metabolic genes in these samples and observed a dysregulated expression of some key glycolytic, fatty acid, and amino acid metabolic genes compared to the matched and non-matched controls. This suggests the role of the dysbiotic virome in regulation of the metabolic activities of breast cancer cells.

Some genes encoded by Parapoxviruses have sequence homology to that of their human counterpart and one of the most extensively studied encoded gene is the VEGF homolog (VEGF-E). Previous reports suggested that these VEGF homologs may bind to human VEGFR2 and trigger downstream signaling^74^. Therefore, we were interested in specifically studying the response of the TNBC cells to VEGF-E homolog expression. We were also curious to see the effect of these VEGF-E homologs on normal cells. We transduced MDA-MB-231 (TNBC cell line) and HMEC (normal breast epithelial cells) with the three different homologs of the Parapoxvirus-encoded VEGF-E and observed that the VEGF-E transduced cells showed higher growth rate and cell proliferation compared to the cells that do not express the homologs. Moreover we also found that the viral VEGF-E are secreted (SI Fig. 3C) and these secreted VEGF-E has the potential to induce a proliferative phenotype in the non-transfected cells (SI Fig. 4). Notably, VEGF-E transfected cells were also altered in their expression of metabolic genes and was quite comparable to that of the tumor samples examined. The effect of VEGF-E homologs on primary HMEC cells was also more prominent compared to that of the MDA-MB-231 cells. This is probably due to the fact that MDA-MB-231 cells is an already transformed cancer cell line with higher proliferation rates, with altered metabolism compared to the normal HMEC cells. Therefore, we would expect that the effect of the VEGF-E homologs would be more significant in the normal HMEC cells. There are previous reports that viral VEGF-E can stimulate cellular VEGF-A production^43^. To determine if the viral VEGF-E is the main driving factor for the enhanced proliferation, we have knocked down the cellular VEGF-A by shRNA and transfected those cells with the viral VEGF-Es and compared the cellular proliferation with the un-transfected cells (SI Fig. 5). Our data clearly showed that the secreted VEGF-E can induce a proliferative potential to non-transfected cells independent of the cellular VEGF-A.

As human VEGFA can activate the PI3K pathway^75^, we were interested in seeing whether the VEGF-E homologs had a similar effect. We found the VEGF-E homologs triggered the PI3K-AKT pathway and altered expression of FoxO1 transcription factor. FoxO1 is a critical transcription factor that not only controls cellular proliferation but is also responsible for regulating the transcription of a number of metabolic genes^76^, maintains homeostasis between metabolic pathways, and is also functionally noted as a tumor suppressor^77^. FoxO1 promotes gluconeogenesis, which typically shuttles TCA intermediates to form glucose^78,79^. Cancer cells, however, follow the opposite path and will avoid oxidative phosphorylation^18,80^. During uncontrolled proliferation FoxO1 is shuttled back to the cytoplasm where it is degraded^60^. We found that the VEGF-E homologs exclude FoxO1 from the nucleus and sequestered it to the cytoplasm. Therefore this would promote the glycolytic pathway activity by downregulating gluconeogenesis, which in turn promotes cell proliferation.

**Fig. 7:**
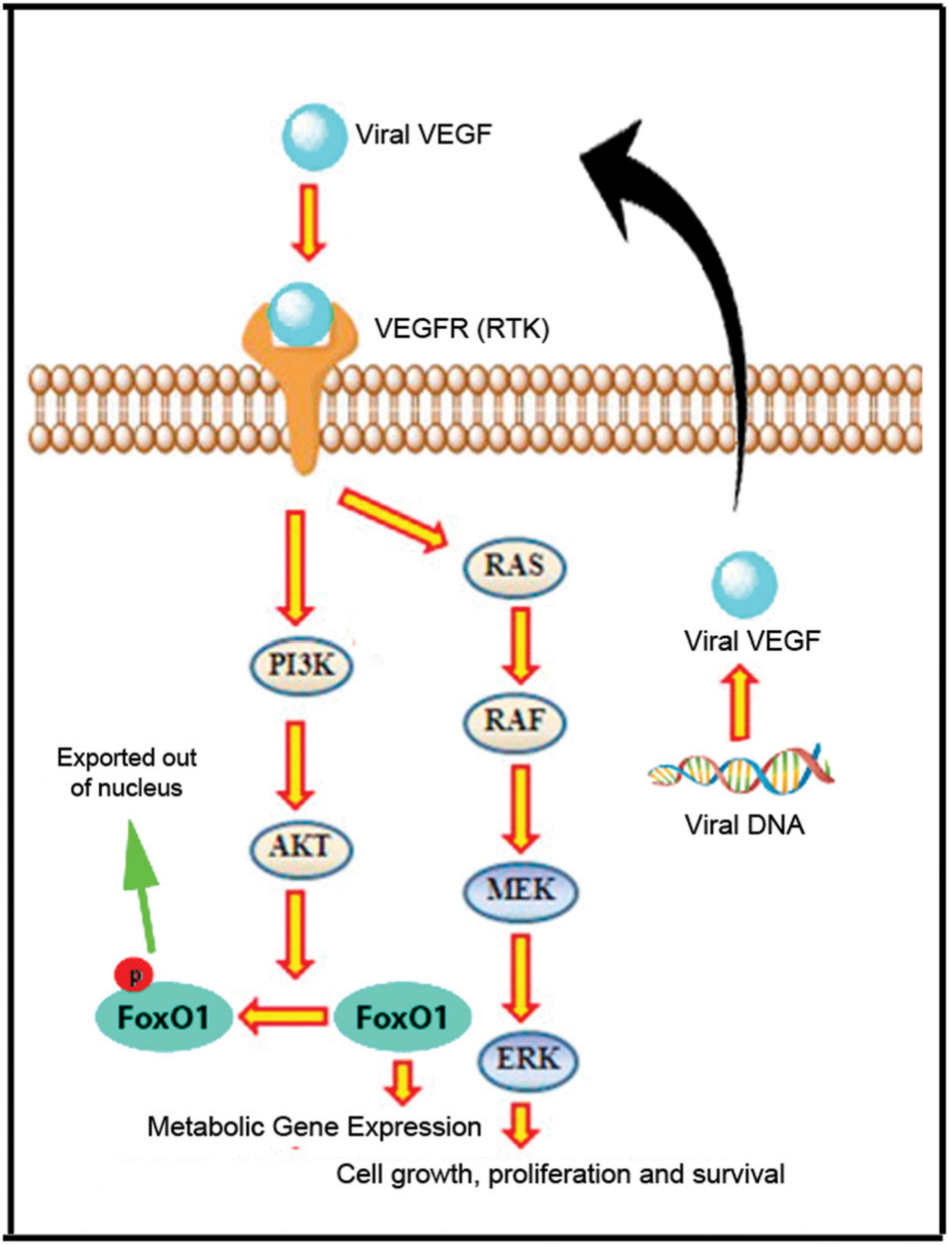
Schematic representation showing VEGF-E modulating of cell proliferation and metabolism. Parapoxvirus, one of the dsybiotic microbiome in the breast cancer cells, encodes viral VEGF-E that acts through the VEGFR and triggers PI3K-AKT and MAPK-ERK pathway. Activated Erk and AKT pathway promotes cell growth, proliferation, and survival. In addition, activated AKT phosphorylates FoxO1 and shuttles its out of the nucleus where it is subsequently degraded. FoxO1 is regarded as tumor suppressor and is linked to expression of different metabolic genes. VEGF-E induced quenching and degradation of FoxO1 facilitates cellular proliferation with altered metabolism.

Inhibition of the PI3K pathway counteracts the effect of the VEGF-E homologs through induction of FoxO1 levels inside the nucleus. This restored the metabolic gene expression levels similar to the cells which do not express the VEGF-E homologs. These results strongly suggested that the Parapoxvirus-encoded VEGF-E acts through the VEGFR2, thus upregulating the VEGFR2 expression in both MDA-MB-231 and HMEC cells (SI Fig. 6) leading to the upregulation of both the PI3K-AKT and ERK-MAPK pathways through sequestration of the FoxO1 transcription factor and altered the metabolic status of the cells (Fig. 7).

Our study provides some initial insights into the action of secretory factors (VEGF-E homologs) encoded by agents associated with a dysbiotic microbiome, which affects proliferation and altered metabolism in tumor cells. Our results also suggest that these VEGF homologs can also induce proliferation, and an altered metabolic phenotype in the normal cells as evident from our HMEC results. Here, we selected one of a plethora of factors from the dysbiotic microbiome in TNBC and dissected its potential role in cancer progression. These in vitro studies highlight the potential strategies utilized by microbial agents to survive and provide mutual benefits for host cells. However, in an in vivo microenvironment there are likely to be other factors produced by other agents in the microbiota that may also play a crucial role in disease pathogenesis. We also should stress that these findings do not directly demonstrate that these parapox viruses are directly responsible for TNBC but the DNA elements from these agents encodes proteins that contributes to the proliferative phenotype of the TNBC. Clearly, additional studies are necessary to further examine the activities of these other agents and their contributions to the disease.

## Supplementary information

Description of additional supplementary files

Supplementary Figure Legends

Supplementary Table 1

Supplementary Table 2

Supplementary Table 3

Supplementary Table 4

Supplementary Table 5

Supplementary Figure 1

Supplementary Figure 2

Supplementary Figure 3

Supplementary Figure 4

Supplementary Figure 5

Supplementary Figure 6
