# Supplementary material for: Vascular endothelial growth factor encoded by Parapoxviruses can regulate metabolism and survival of triple negative breast cancer cells
Source: Cell Death Dis. 2020 Nov 20;11(11):996. doi: 10.1038/s41419-020-03203-4 (PMC7679371; doi:10.1038/s41419-020-03203-4)
Supplement: Supplementary file 1 — Description of additional supplementary files [file 41419_2020_3203_MOESM1_ESM.docx]

**Description of additional supplementary files**

**Supplementary Table legends**

**Supplementary Table 1:** Sequences for real time PCR primers against different Parapox virus used to validate the PathoChip results.

**Supplementary Table 2:** Real time PCR primers against different VEGF.

**Supplementary Table 3:** Sequences for real time PCR primers for different metabolic genes.

**Supplementary Table 4:** Details of antibodies used in the study.

**Supplementary Table 5:** The source data for the manuscript (Excel File)
