# Supplementary material for: Vascular endothelial growth factor encoded by Parapoxviruses can regulate metabolism and survival of triple negative breast cancer cells
Source: Cell Death Dis. 2020 Nov 20;11(11):996. doi: 10.1038/s41419-020-03203-4 (PMC7679371; doi:10.1038/s41419-020-03203-4)
Supplement: Supplementary file 2 — Supplementary Figure Legends [file 41419_2020_3203_MOESM2_ESM.docx]

**Supplementary Figure Legend**

**SI Figure 1: Validation of the PathoChip result using specific primers for ORFV, PCPV and BPSV.**

50 ng of whole transcriptome amplicon (WTA) was used for quantitative PCR reaction using primers specific for the Parapoxvirus was used to validate the presence or absence of poxvirus in the tissue samples. DNA isolated from the strains of virus were used as positive control and DNA from MDA-MB231 cells were used as negative control. A heatmap for presence or absence is presented. The real-time PCR experiments were performed in triplicates, with an experimental repeat for each gene.

**SI Figure 2: Detection of Parapox virus VEGF-E homologs in tissue samples.**

50 ng of whole transcriptome amplicon (WTA) was used for quantitative PCR reaction using primers specific for the Parapoxvirus encoded VEGF-E was used to validate the presence or absence of poxvirus in the tissue samples. DNA isolated from the strains of virus were used as positive control and DNA from MDA-MB-231 cells were used as negative control. A heatmap for presence or absence is presented. Human VEGFA was selected as a control. The real-time PCR experiments were performed in duplicates, with an experimental repeat for each gene.

**SI Figure 3: Validation of the VEGF-E clones in pLVX vector and its expression and secretion in MDA- MB-231 and HMEC cells.**

A. The viral VEGF-Es were cloned in pLVX-AcGFP vector and were transfected in MDA-MB-231 and HMEC cells. To check the cloning specific primers were used to amplify the cloned DNA and was resolved in agarose gel. B. To check the expression of the VEGF-E protein fused with GFP western blot was performed using antibodies against GFP. C. ELISA assay was performed from the cell supernatant using anti-GFP antibody and quantified using Cytation 5. The experiments were performed in triplicates with two experimental repeats.

**SI Figure 4: Secreted VEGF-E alters cell proliferation.**

A. Cell culture supernatant was collected from 24 h culture of VEGF-E transfected MDA-MB-231 and HMEC cells, mixed with equal proportion with fresh media and MDA-MB-231 and HMEC cells respectively were cultured using that media. B. Cell culture supernatant was collected from 24 h culture of VEGF-E transfected MDA-MB-231 and HMEC cells, treated with protein a/g tagged anti-GFP neutralizing antibody for 4 h, centrifuged and the supernatant was mixed in equal proportion with fresh media and MDA-MB-231 and HMEC cells were cultured using that media. Cellular proliferation was measured by MTT assay. All the experiments were performed in triplicates with two experimental repeats.

**SI Figure 5: Role of cellular VEGF-A on viral VEGF-E mediated cellular proliferation.**

A. Knockdown of the cellular VEGF-A verified by western blot. B. The proliferation of the KD cells was assessed by MTT assay. The experiments were performed in triplicates with two experimental repeats.

**SI Figure 6: Upregulation of VEGFR2 in MDA-MB-231 and HMEC cells expressing the viral VEGF-E**

MDA-MB-231 and HMEC cells stably expressing viral VEGF-E or mock were stained with VEGFR2 antibody tagged with APC and the change of expression of VEGFR2 was measured by flow cytometry and the mean fluorescence intensity was calculated using FlowJo software and represented as bar plots. The experiment was performed in duplicates.
