# Supplementary material for: Vascular endothelial growth factor encoded by Parapoxviruses can regulate metabolism and survival of triple negative breast cancer cells
Source: Cell Death Dis. 2020 Nov 20;11(11):996. doi: 10.1038/s41419-020-03203-4 (PMC7679371; doi:10.1038/s41419-020-03203-4)
Supplement: Supplementary file 3 — Supplementary Table 1 [file 41419_2020_3203_MOESM3_ESM.docx]

**Supplementary Table 1:** Real time PCR primers against different Parapox virus used to validate the PathoChip results:

| Organism | Ref Seq No | Forward Primer | Reverse Primer | Position |
| --- | --- | --- | --- | --- |
| ORFV | NC_005336 | ATGACGCGCGGGTAAAAGCACA | TGACGCTCATGGACAGCCTGGA | 91147-91465 |
| BPSV | NC_005337 | CACGTGATGCTGAGCAACGGGT | TTGTACGCCTCCAGCAGCTCCT | 72974-73074 |
| PCP | NC_013804 | ACTCATCTTGACGGAACCTGGG | CTATGGCGCCTAGGCTGGTAAT | 119908-120133 |
