# Supplementary material for: Vascular endothelial growth factor encoded by Parapoxviruses can regulate metabolism and survival of triple negative breast cancer cells
Source: Cell Death Dis. 2020 Nov 20;11(11):996. doi: 10.1038/s41419-020-03203-4 (PMC7679371; doi:10.1038/s41419-020-03203-4)
Supplement: Supplementary file 4 — Supplementary Table 2 [file 41419_2020_3203_MOESM4_ESM.docx]

**Supplementary Table 2: Real time PCR primers against different VEGF**

| Gene Name | Forward | Reverse |
| --- | --- | --- |
| VEGFA | GCAGAATCATCACGAAGTGGTG | GGTACTCCTGGAAGATGTCCAC |
| ORFV-VEGF-E | ATGAAGTTGCTCGTCGGCATA | TGTCCGCGTTCAGCAGATACT |
| PCPV-VEGF-E | CGGTAGCGTTGTTGATATGTA | CTAACCAACTACTTAACGATGA |
| BPSV-VEGF-E | CGATGAAAGTTTGGAATGTGTT | GCACTCACATTTGTTATGTTGT |
