# Supplementary material for: Vascular endothelial growth factor encoded by Parapoxviruses can regulate metabolism and survival of triple negative breast cancer cells
Source: Cell Death Dis. 2020 Nov 20;11(11):996. doi: 10.1038/s41419-020-03203-4 (PMC7679371; doi:10.1038/s41419-020-03203-4)
Supplement: Supplementary file 5 — Supplementary Table 3 [file 41419_2020_3203_MOESM5_ESM.docx]

**Supplementary Table 3:** Real time PCR primers against different metabolic genes:

| Gene Name | Gene Abbreviation | Forward | Reverse |
| --- | --- | --- | --- |
| Phospho Fructkinase 2 | PFK2 | TACGACTTCTTTCGGCATGA | CTCCTCTCCCGGGTTGTATT |
| Pyruvate Kinase M | PKM | AGGCAGCCATGTTCCAC | TGCCAGACTCCGTCAGAACT |
| Lactate Dehydrogenase | LDH | GACCTACGTGGCTTGGAAGA | TCCATACAGGCACACTGGAA |
| Bisphosphoglycerate Mutase | BPGM | CTGGAAGAGCTAGGCCAGGA | GCAGATGGCTTTGAATCATG |
| Phosphoglycerate kinase | PGK1 | CCTGCTGGAGAACCTCCGCT | AGTGAAGCTCGGAAAGCTTC |
| Enolase 1 | ENO1 | GCCATGCAGGAGTTCATGAT | CCATATTTCTCCTTGATGAC |
| Fructose bisphosphatase | FBP | GCTACGCACTGTATGGCAGTG | ATCTTCACATCCTTGTCCACC |
| Glucose-6-phosphate dehydrogenase | G6PD | GGTGCAGGCCAACAATGTGG | GGACCCGCGGGGCACCGTGG |
| Pyruvate dehydrogenase complex E1 | PDHA1 | GTGATGGTCAGGAAGCTTGC | GCCCCGGGTGAAAGTAAAGC |
| Hexokinase 1 | HK1 | GACTGGACCGTCTGAATGT | ACAGTTCCTTCACCGTCTGG |
| Glucose Transporter 1 | GLUT1 | AAGCTGACG GGTCGCCTCATG | CTCTCCCCATAGCGGTGGACC |
| ATP Citrate Lyase | ACLY | GGAGTTTGTGAACAAGATGA | ATCTGCACTCGCATGTCTGG |
| Argininosuccinate Lyase | ASL | CGACACTATCCGTGCGGC | ACCAATGAGCTCCTTCAGGC |
| Arginase2 | ARG2 | CTATCAGCACTGGATCTTGTTG | GGGAGTAGGAAGTTGGTCATAG |
| N-acetylglutamate synthase | NAGS | CAGACTGCCACTCTTGGGG | GGCAAAGGCCAGACTGGATA |
| Nitric Oxide synthase | NOS2 | TCCAAGGTATCCTGGAGCGA | CAGGGACGGGAACTCCTCTA |
| Branched-chain amino acid aminotransferase | BCAT | GGGCTACGACCCTTGGGAT | CCCTCACAGCAGAGCGATAC |
| Fatty Acid synthase | FAS | AGGAACTCCCCTCATCTCCC | TTGCCGTTCTCTGACACCTC |
| Carnitine palmitoyltransferase 1A | CPT1A | CATACGAGGCCTCCATGACC | TTTCCAGCCCAGCACATGAA |
| Acetyl-CoA carboxylase | ACC1 | TCTTGGCCTTTTCCCGGTC | CTCTGAAGCCCAAAGAGGGG |
| 3-Hydroxy-3-Methylglutaryl-CoA Synthase 1 | HMGCS | TTGTGCCCGAAGGAGGAAAC | GCCGCCCAATGCAATCATAG |
| Acetyl-CoA Acetyltransferase 1 | ACAT | GAGCCGATACTCAGCCCTCT | GGAGCTTGTCCTTCACCTCC |
| 18S ribosomal RNA | 18S | GTAACCCGTTGAACCCCATT | CCATCCAATCGGTAGTAGCG |
