# Supplementary material for: Vascular endothelial growth factor encoded by Parapoxviruses can regulate metabolism and survival of triple negative breast cancer cells
Source: Cell Death Dis. 2020 Nov 20;11(11):996. doi: 10.1038/s41419-020-03203-4 (PMC7679371; doi:10.1038/s41419-020-03203-4)
Supplement: Supplementary file 6 — Supplementary Table 4 [file 41419_2020_3203_MOESM6_ESM.docx]

| **Antibody name** | **Company** | **Catalog Number** |
| --- | --- | --- |
| Ki67 | Santa Cruz Biotechnology | Sc-23900 |
| PCNA | Santa Cruz Biotechnology | Sc-25280 |
| GAPDH | Santa Cruz Biotechnology | Sc-47724 |
| p-ERK | Santa Cruz Biotechnology | Sc-7383 |
| ERK | Cell Signaling Technologies | 9102 |
| p-MEK | Santa Cruz Biotechnology | Sc-136542 |
| P-RAF | Santa Cruz Biotechnology | Sc-271929 |
| pI3K p110 | Santa Cruz Biotechnology | Sc-8010 |
| AKT | Santa Cruz Biotechnology | Sc-1618 |
| p-AKT | Cell Signaling Technologies | 5102 |
| FOXO1 | Santa Cruz Biotechnology | Sc-374427 |
| Anti-GFP | Santa Cruz Biotechnology | Sc-8334 |
| Anti BrdU-PE | Invitrogen, Thermo Fisher | 12-5071-42 |
| Anti-VEGFR2-Alexa 647 | Santa Cruz Biotechnology | Sc-6251-AF647 |
| H2A | Santa Cruz Biotechnology | Sc-10807 |

**Supplementary Table 4: Details of antibodies used in the study**
