# Supplementary figures and images for: Vascular endothelial growth factor encoded by Parapoxviruses can regulate metabolism and survival of triple negative breast cancer cells
Source: Cell Death Dis. 2020 Nov 20;11(11):996. doi: 10.1038/s41419-020-03203-4 (PMC7679371; doi:10.1038/s41419-020-03203-4)

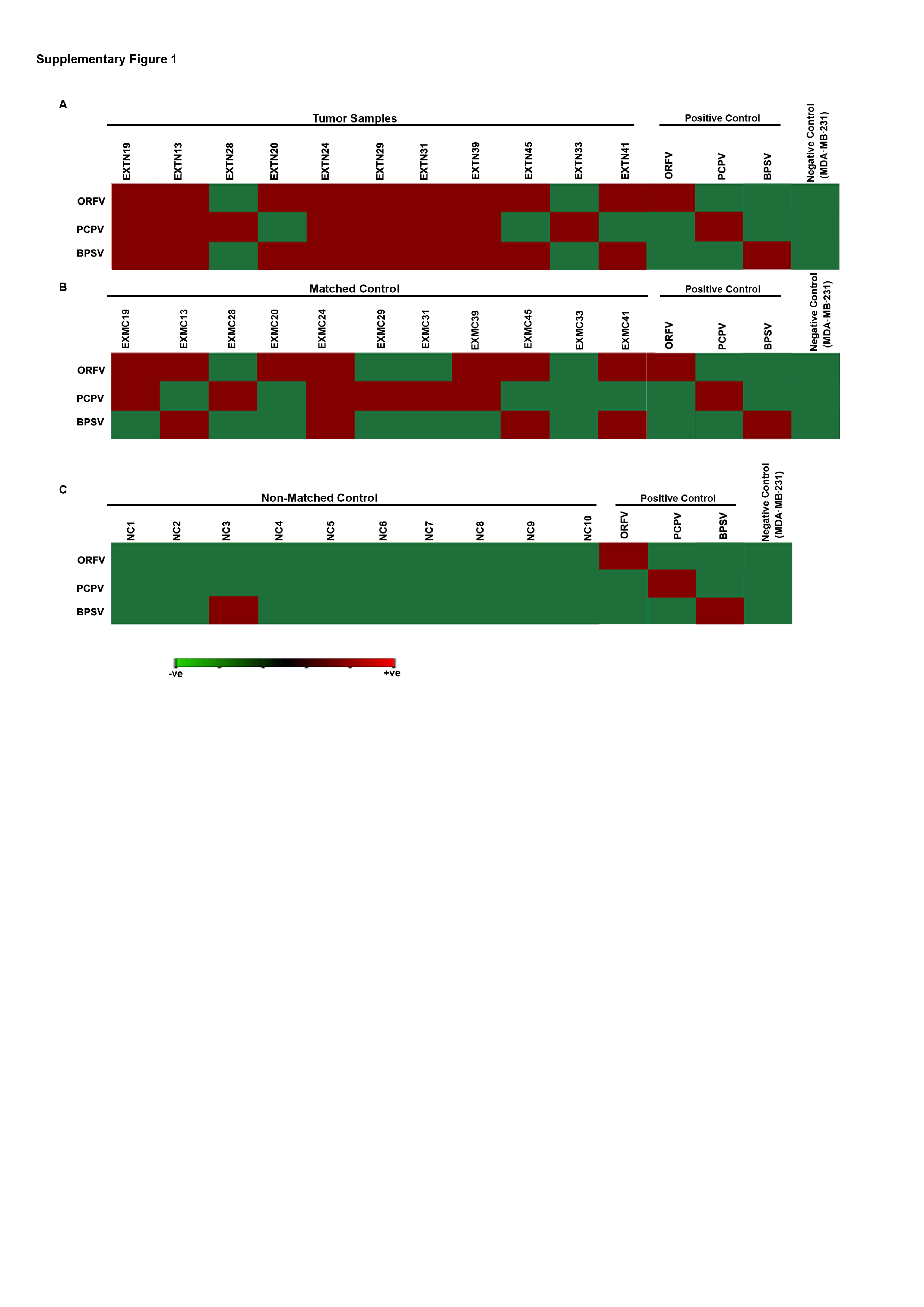

Supplement: Supplementary file 8 — Supplementary Figure 1 [file 41419_2020_3203_MOESM8_ESM.tif]

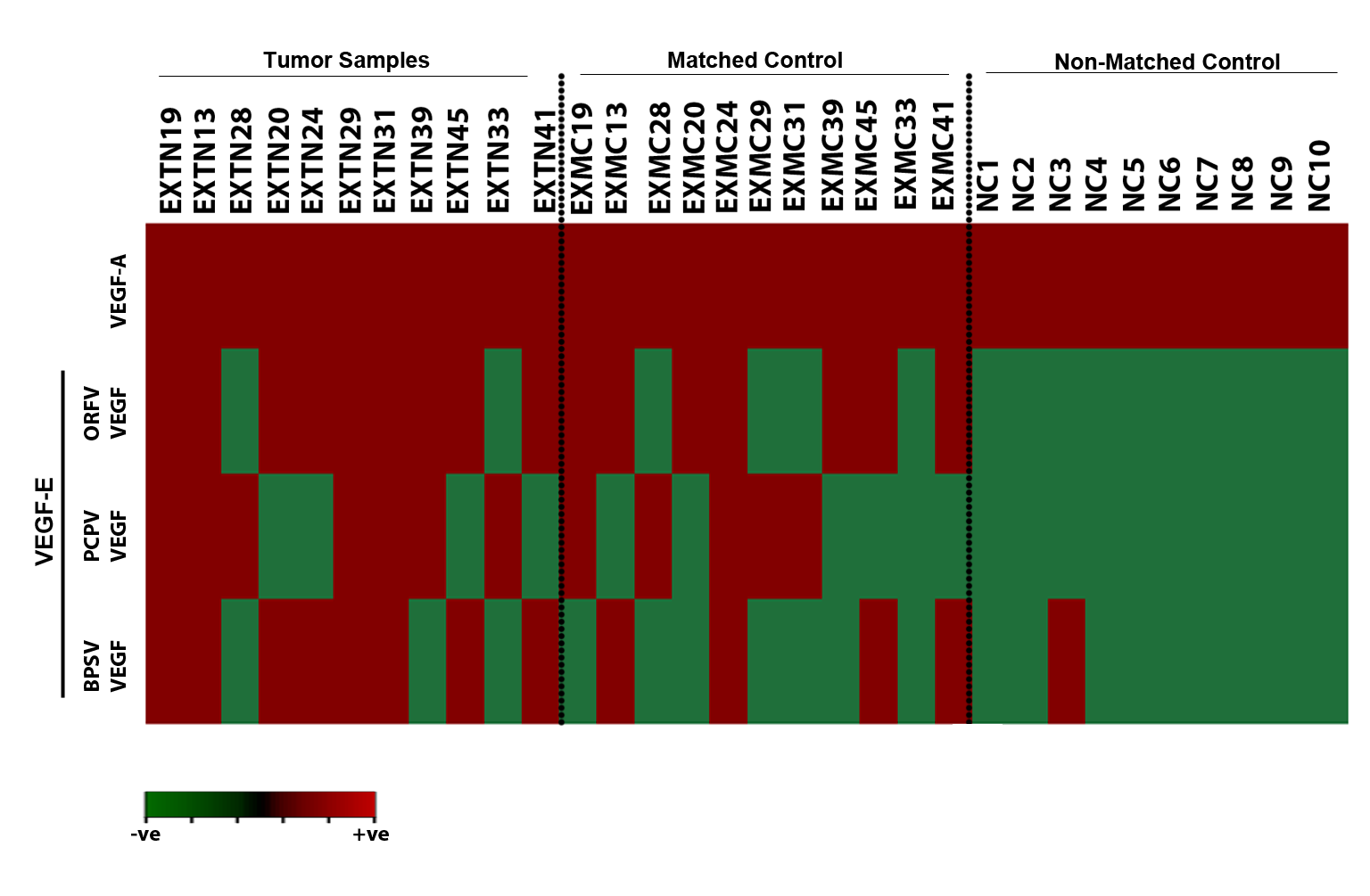

Supplement: Supplementary file 9 — Supplementary Figure 2 [file 41419_2020_3203_MOESM9_ESM.tif]

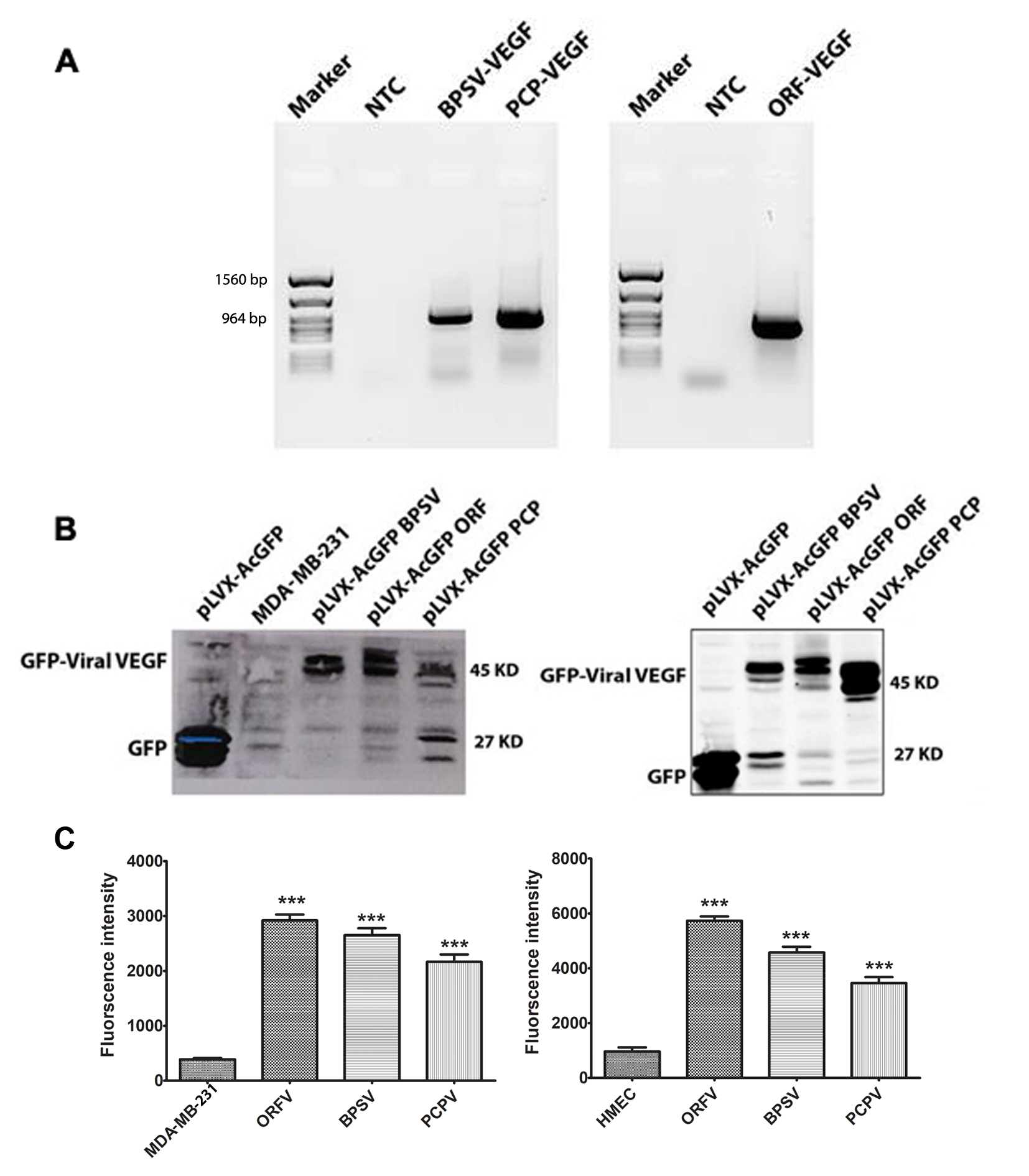

Supplement: Supplementary file 10 — Supplementary Figure 3 [file 41419_2020_3203_MOESM10_ESM.tif]

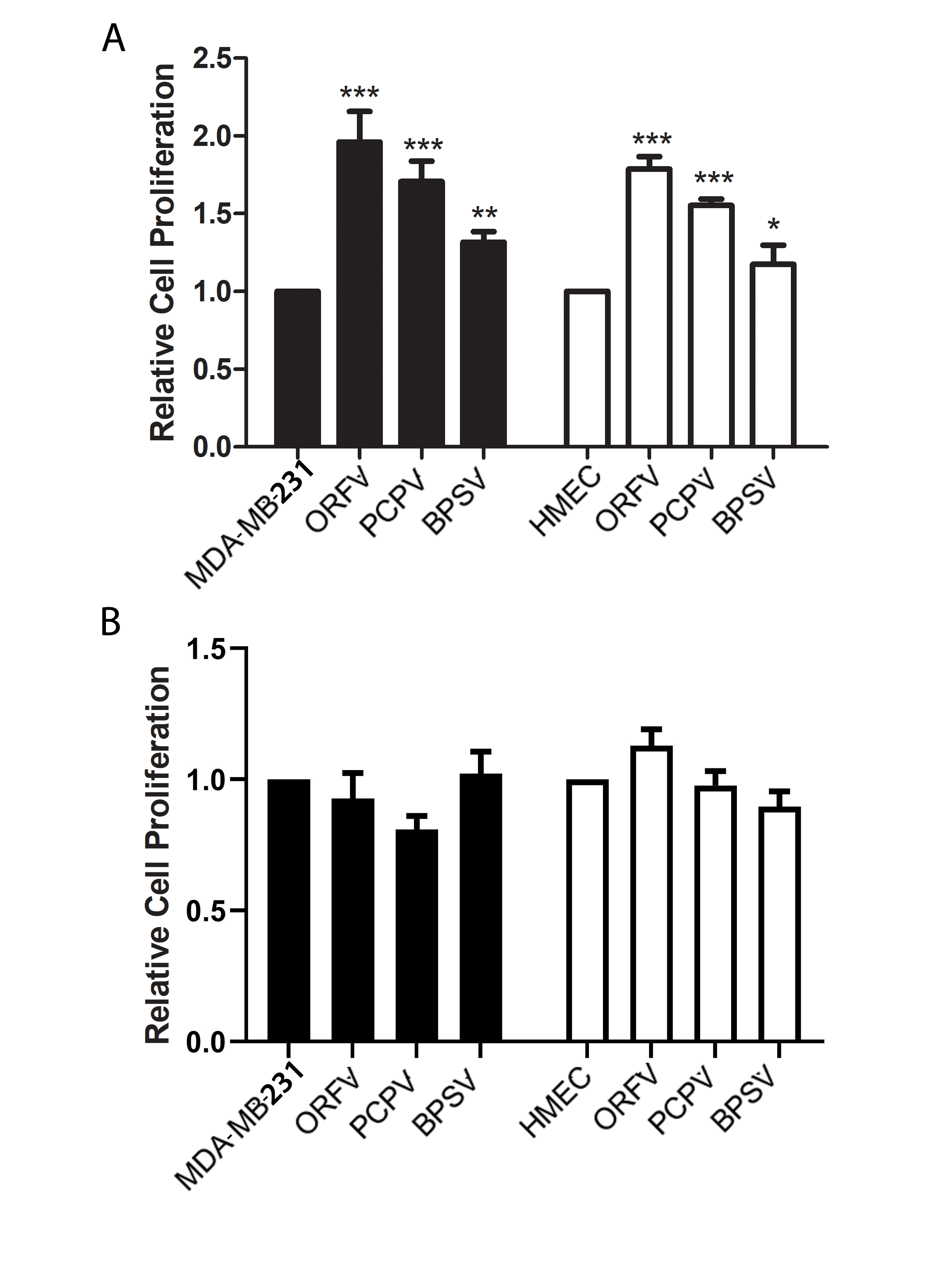

Supplement: Supplementary file 11 — Supplementary Figure 4 [file 41419_2020_3203_MOESM11_ESM.tif]

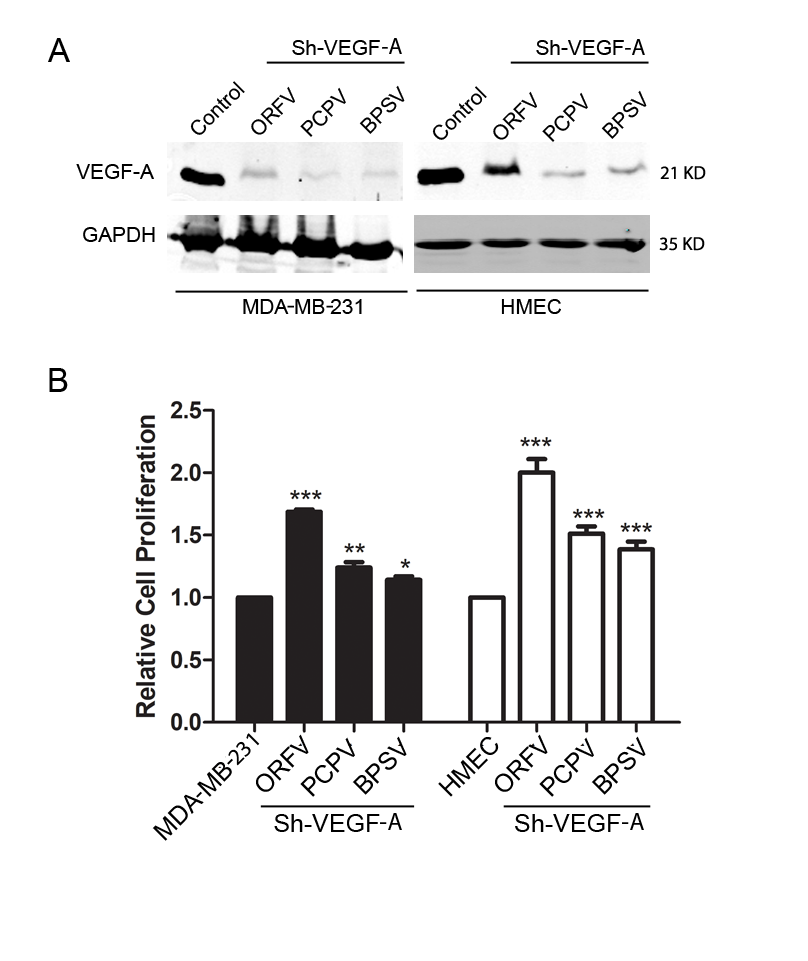

Supplement: Supplementary file 12 — Supplementary Figure 5 [file 41419_2020_3203_MOESM12_ESM.tif]

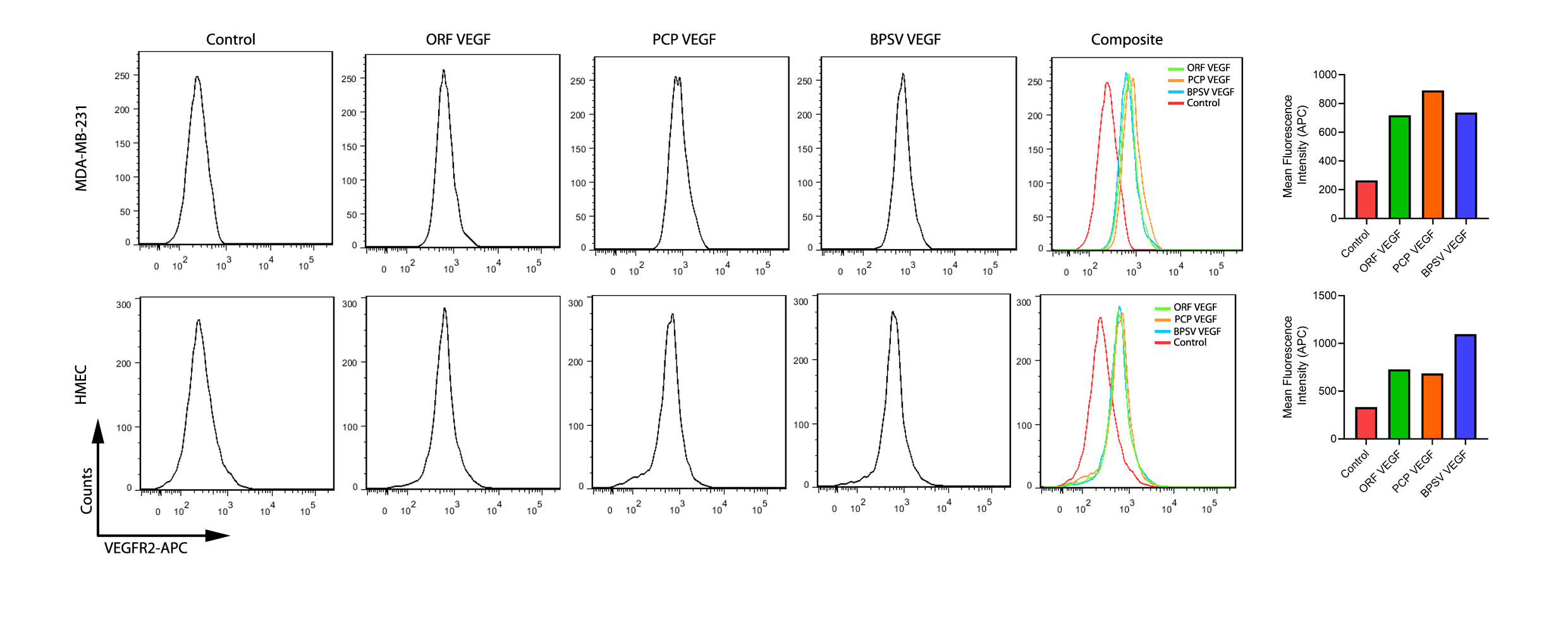

Supplement: Supplementary file 13 — Supplementary Figure 6 [file 41419_2020_3203_MOESM13_ESM.tif]
